# Laparoscopic approach for surgical treatment of pleuroperitoneal communication interfering with peritoneal dialysis: a case report

**DOI:** 10.1186/s40792-021-01228-1

**Published:** 2021-09-28

**Authors:** Satoko Yorinaga, Takehiro Maki, Noriko Kawai, Hiroyuki Kaneko, Kenjiro Misu, Hitoshi Inomata, Makoto Omi, Satoshi Hirano

**Affiliations:** 1 Department of Surgery, Kushiro Red Cross Hospital, Kushiro, Japan; 2 Department of Gastroenterological Surgery II, Hokkaido University Hospital, Sapporo, Japan

**Keywords:** Combined thoraco- and laparoscopic surgery, Pleuroperitoneal communication, Peritoneal dialysis

## Abstract

**Background:**

Pleuroperitoneal communication is a rare disorder that interferes with peritoneal dialysis. Although favorable results of thoracoscopic fistula closure have been reported, there are some cases in which the fistulas cannot be identified by thoracoscopy and the patients are forced to switch to hemodialysis.

**Case presentation:**

We present two cases of pleuroperitoneal communication in which diaphragmatic fistulas could not be identified thoracoscopically, but could be identified laparoscopically. Patient 1 had difficulty continuing peritoneal dialysis 9 months after its introduction due to right pleural effusion. Although we could not detect the fistula thoracoscopically, we could laparoscopically identify the fistula in the center of the tendon of the right diaphragm and closed the site from the thoracic side. Patient 2 developed dyspnea due to right pleural effusion 6 months after the introduction of peritoneal dialysis. We could not find the fistulas with a thoracoscopic approach, but could identify multiple diaphragmatic fistulas with a laparoscopic approach and close the sites from the thoracic side.

**Conclusion:**

In the surgical treatment of pleuroperitoneal communication, diaphragmatic fistulas can be identified laparoscopically even when thoracoscopic observation fails to find any fistulas.

## Background

Pleuroperitoneal communication is a rare condition, which often forces patients with peritoneal dialysis to switch to hemodialysis due to the accumulation of pleural effusion [1, 2]. Although the thoracoscopic approach has been reported to be useful in the surgical treatment of pleuroperitoneal communication [3], in some cases, the diaphragm defects cannot be confirmed with a thoracoscope [4]. We present two cases of pleuroperitoneal communication in which the diaphragm defects were not identified with a thoracoscope but with a laparoscope.

## Case presentation

### Patient 1

**Fig. 1:**
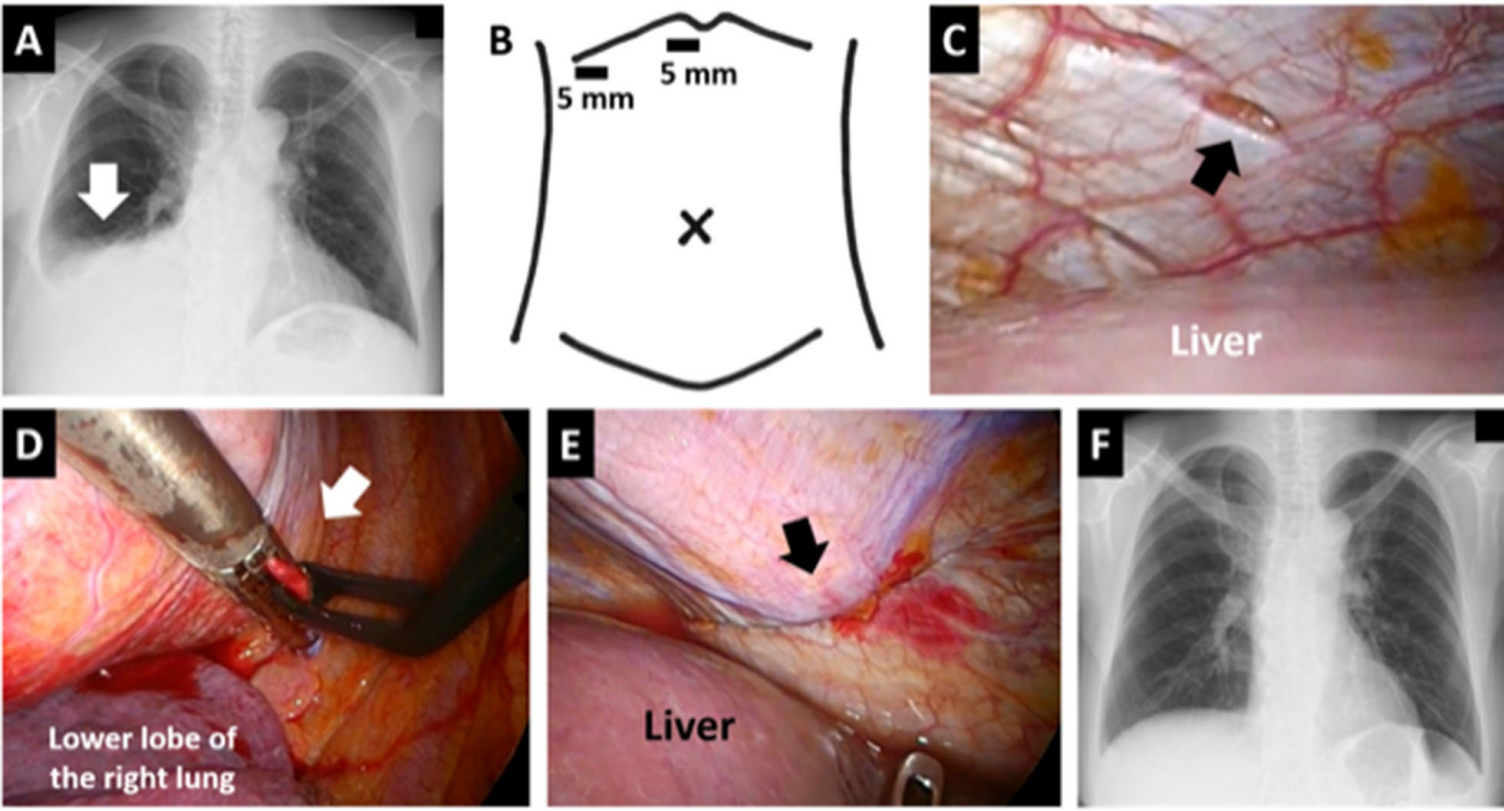
Diagnosis and treatment for pleuroperitoneal communication in Patient 1. **A** Chest radiograph taken 2 months after the introduction of peritoneal dialysis. An arrow shows right pleural effusion. **B**. Port positions in the laparoscopic approach. **C** Laparoscopic view of the diaphragmatic fistula. An arrow shows a 2-mm-sized fissure in the central tendon of the right diaphragm over the liver. **D** A thoracic view of the closure of the diaphragmatic fistula using an automatic suture device. An arrow shows the grasped site of the right diaphragm, including the fistula. **E** Laparoscopic view of the closed site. The arrow shows the suture line. **F** Chest radiograph taken 3 months after the surgery

A 63-year-old woman with chronic renal failure due to polycystic kidney disease and diabetes started peritoneal dialysis. Two months after the introduction of peritoneal dialysis, a chest radiograph showed right pleural effusion without any symptoms (Fig. 1A). The pleural effusion disappeared with diuretics, and peritoneal dialysis was continued. However, 9 months after the introduction, poor water removal interfered with peritoneal dialysis and computer tomography showed recurrence of the right pleural effusion. We diagnosed that right pleural effusion due to right pleuroperitoneal communication, disturbed peritoneal dialysis, and performed surgical treatment 11 months after the introduction of peritoneal dialysis. Considering the possibility of using a laparoscopy, we sterilized not only the chest, but also the upper abdomen before skin incision. We first attempted a right thoracoscopic approach with four intercostal ports in the left lateral decubitus position deflating the right lung and could not identify any defects in the diaphragm at all, although 2,000 mL of peritoneal dialysate mixed with 40 mg of indigo carmine was injected into the abdominal cavity or CO_2_ was insufflated with an abdominal pressure of 10 cmH_2_O from a peritoneal dialysis catheter. Next, we attempted a laparoscopic approach using two ports with a diameter of 5 mm in the upper right abdomen (Fig. 1B) in the semi-left lateral decubitus position and found a 2-mm-sized fissure in the central tendon of the right diaphragm (Fig. 1C). We laparoscopically grasped the fistula with the surrounding tissue and thoracoscopically sutured and closed the gripped part using an automatic suture device (Fig. 1D, E). The closure was reinforced with polyglycolic acid sheets and fibrin glue under thoracoscopy. A drainage tube was placed in the right thoracic cavity. No postoperative complications occurred. The patient continued peritoneal dialysis without recurrent right pleural effusion for several months (Fig. 1F). Five months after the surgery, intra-abdominal infection developed, the peritoneal dialysis catheter was removed, and the patient was switched to hemodialysis.

### Patient 2

**Fig. 2:**
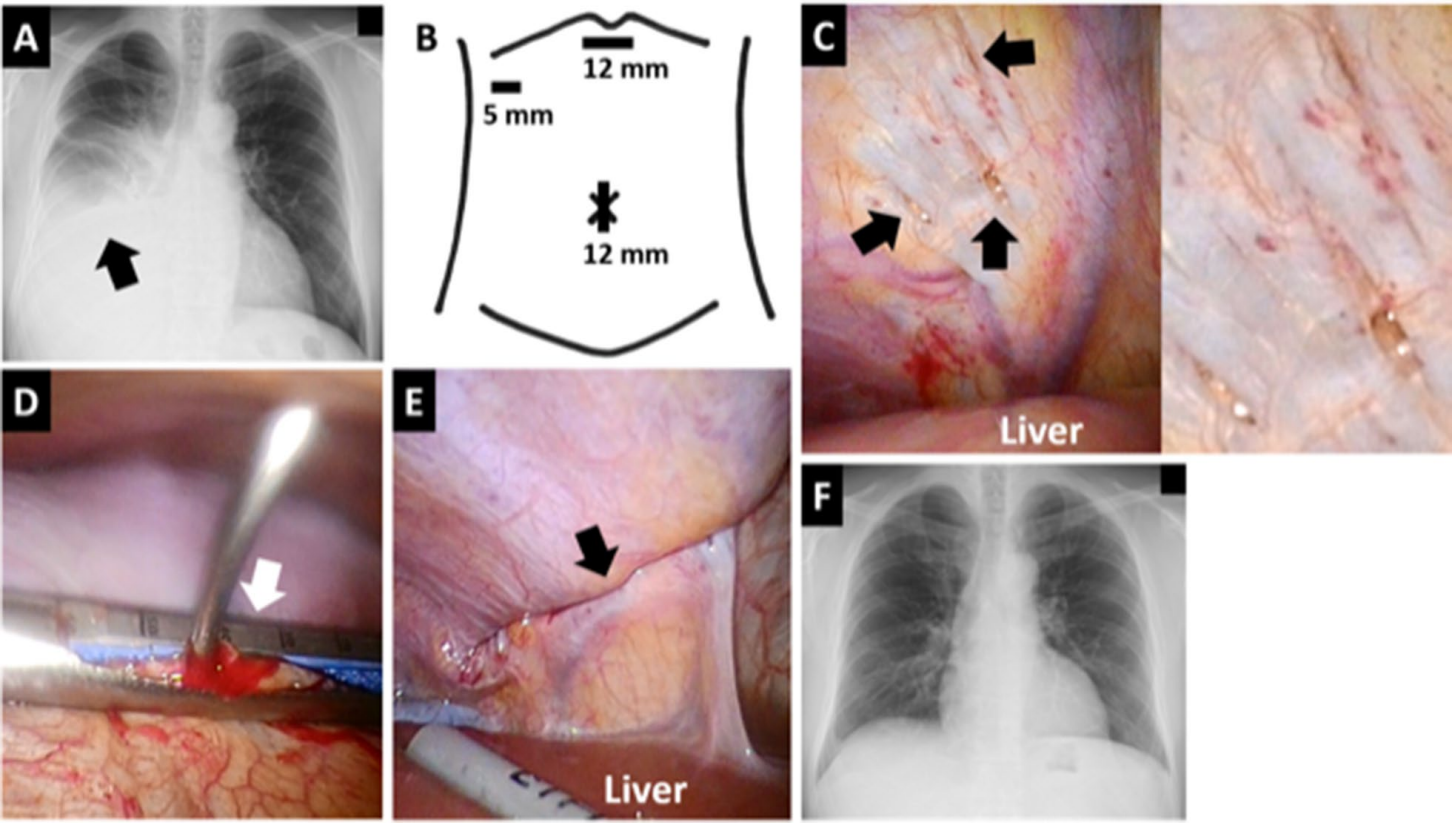
Diagnosis and treatment for pleuroperitoneal communication in Patient 2. **A** Chest radiograph taken 6 months after the introduction of peritoneal dialysis. An arrow shows right pleural effusion. **B** Port positions in the laparoscopic approach. **C** Laparoscopic view of the diaphragmatic fistulas. Arrows show three fissures 1–2 mm in size in the central tendon of the right diaphragm over the liver. The image on the right is a magnified view of the fissures. **D** A thoracic view of the closure of the diaphragmatic fistulas using an automatic suture device. An arrow shows the grasped site of the right diaphragm, including all the fistulas. **E** Laparoscopic view of the closed site. The arrow shows the suture line. **F** Chest radiograph taken 10 months after surgery

A 38-year-old man with chronic renal failure due to immunoglobulin A nephropathy started peritoneal dialysis. Six months after the introduction of peritoneal dialysis, the patient suddenly experienced dyspnea, and a chest radiograph showed right pleural effusion (Fig. 2A). We diagnosed that right pleural effusion due to right pleuroperitoneal communication caused the symptom and performed surgical treatment 7 months after the introduction of peritoneal dialysis. Considering the possibility of using a laparoscopy, we sterilized not only the chest, but also the upper abdomen before skin incision. We first tried a right thoracoscopic approach with three intercostal ports in the left lateral decubitus position deflating the right lung and could not identify any diaphragmatic defects at all, although 2000 mL of peritoneal dialysate mixed with 40 mg of indigo carmine was injected into the abdominal cavity from a peritoneal dialysis catheter. Next, we tried a laparoscopic approach using three ports with a diameter of 12 mm in the umbilicus, 12 mm in the epigastrium, and 5 mm in the upper right abdomen (Fig. 2B) in the semi-left lateral decubitus position. We found three fissures 1–2 mm in size in the central tendon of the right diaphragm (Fig. 2C). We grasped all of them laparoscopically and sutured and closed the gripped part using an automatic suture device thoracoscopically (Fig. 2D, E). The closure was thoracoscopically reinforced with coagulation factor XIII plus fibrinogen. A drainage tube was placed in the right thoracic cavity. No postoperative complications occurred. Ten months after the surgery, right pleural effusion was not observed (Fig. 2F), and the patient continued peritoneal dialysis without any problems.

## Conclusions

Pleuroperitoneal communication is a rare condition encountered by 1.6–1.9% of patients with chronic renal failure undergoing peritoneal dialysis, and 50% of them are forced to switch to hemodialysis [1, 2]. Fifty-four percent of patients are female and 74% of the patients suffer from cough, chest pain, and dyspnea, while the others are asymptomatic [1]. Eighty-eight percent of the disease occurs on the right side and 4% of the disease occurs on both sides [1]. Generally, it is thought that the transfer of peritoneal dialysis fluid through the fistula of the diaphragm to the thoracic cavity causes various symptoms and complicates continued peritoneal dialysis, although pleural effusion can also be caused by lymphatic migration from the abdominal cavity [5]. Therefore, it is considered that proof of diaphragmatic fistulas with a pigment, contrast medium, or isotope is important in the diagnosis, while closure of the fistulas is essential for the treatment of pleuroperitoneal communication [6, 7].

The success rate of non-surgical treatments, such as reducing the amount of injected peritoneal dialysate, temporarily discontinuing peritoneal dialysis, and chemical pleurodesis, was reported to be 54% [1]. The first reported surgical treatment for pleuroperitoneal communication was fistula closure with a polytetrafluoroethylene patch via thoracotomy by Pattison et al. in 1984 [8]. In 1996, Di Bisceglie et al. reported the usefulness of video-assisted thoracoscopic surgery for the disease [3]. Since then, thoracoscopic surgery, which involves closing the diaphragmatic fistula with sutures or automatic suture devices and covering the fistula with a mesh or sheet, has been considered the standard therapy for the disease. Saito et al. reported a 72% success rate with such thoracoscopic surgery [4]. On the other hand, a previous study showed that diaphragmatic fistulas could not be confirmed by thoracoscopy in 28% of cases and the treatment success rate in such cases was only 38% [4].

We herein report two cases of pleuroperitoneal communication in which diaphragmatic fistulas could be confirmed by laparoscopy, although not identified by thoracoscopy. Recently, Manabe et al. have reported laparoscopic approach for pleuroperitoneal communication to emphasize the efficacy of pneumoperitoneum [9]. They could find diaphragmatic fistulas under a thoracoscope by pneumoperitoneum, but we could not find them in the present two cases with that method. Instead, we could find diaphragmatic fistulas by laparoscopic observation. This is the first report on visualization of diaphragmatic fistulas under laparoscopic observation in surgical treatment of pleuroperitoneal communication. In the treatment of pleuroperitoneal communication, laparoscopic observation might be useful in detecting diaphragmatic fistulas if thoracoscopic observation fails and finding possibly missed fistulas in a thoracoscope.

The structures of fistulas in pleuroperitoneal communication may be more visible from the abdominal side than from the thoracic side. The shape of the fistulas was a fissure when viewed from a laparoscope in our two cases. This is in contrast to the small circular holes that were observed from a thoracoscope in other reports [4, 5]. The shape of the fistula observed with a laparoscope may be related to the rate of detection of the fistula with a thoracoscope. Further accumulation of structural information concerning the diaphragmatic fistulas may reveal the etiology of pleuroperitoneal communication.

## Data Availability

Not applicable.
